# Multi-physiological signal fusion for objective emotion recognition in educational human–computer interaction

**DOI:** 10.3389/fpubh.2024.1492375

**Published:** 2024-11-26

**Authors:** Wanmeng Wu, Enling Zuo, Weiya Zhang, Xiangjie Meng

**Affiliations:** ^1^School of International Education and Exchange, Changchun Sci-Tech University, Changchun, China; ^2^School of Education, Changchun Normal University, Changchun, China; ^3^School of Foreign Languages, Jilin Normal University, Changchun, China; ^4^Informatization Center, Changchun Sci-Tech University, Changchun, China

**Keywords:** human–computer interaction, emotion recognition, multi-physiological signals, wearable devices, educational evaluation, artificial intelligence

## Abstract

**Introduction:**

An increasing prevalence of psychological stress and emotional issues among higher education teachers necessitates innovative approaches to promote their wellbeing. Emotion recognition technology, integrated into educational human–computer interaction (HCI) systems, offers a promising solution. This study aimed to develop a robust emotion recognition system to enhance teacher–student interactions within educational HCI settings.

**Methods:**

A multi-physiological signal-based emotion recognition system was developed using wearable devices to capture electrocardiography (ECG), electromyography (EMG), electrodermal activity, and respiratory signals. Feature extraction was performed using time-domain and time-frequency domain analysis methods, followed by feature selection to eliminate redundant features. A convolutional neural network (CNN) with attention mechanisms was employed as the decision-making model.

**Results:**

The proposed system demonstrated superior accuracy in recognizing emotional states than existing methods. The attention mechanisms provided interpretability by highlighting the most informative physiological features for emotion classification.

**Discussion:**

The developed system offers significant advancements in emotion recognition for educational HCI, enabling more accurate and standardized assessments of teacher emotional states. Real-time integration of this technology into educational environments can enhance teacher–student interactions and contribute to improved learning outcomes. Future research can explore the generalizability of this system to diverse populations and educational settings.

## 1 Introduction

In the current epoch, our nation is experiencing a period of substantial societal metamorphosis, defined by sweeping transformations in the realms of economy and society. This period is distinguished by swift changes in social frameworks, ways of living, behavioral norms, and the cultural fabric of our nation, alongside a continuous evolution of societal values (1). The quickening tempo of modern life has resulted in more intricate interpersonal dynamics and heightened social rivalry (2). Such shifts have discreetly amplified the psychological strain on individuals, fostering a widespread sentiment of insecurity and fostering conditions conducive to anxiety, depression, and an array of other mental health concerns. As a result, mental health has become an increasingly pressing concern within society (3).

Institutions of higher learning are instrumental in cultivating future leaders, propelling scientific and technological advancements, and catalyzing societal progress (4). University educators, as indispensable assets, hold a distinct position in society, shaped by their roles and duties (5). They are the cornerstone of fulfilling the missions and obligations of these institutions (6). However, within the wider social context, university educators are confronted with a new array of challenges and pressures, which have triggered a spectrum of psychological issues and incidents (7). This has ignited significant societal concern for the mental wellbeing of those in the teaching profession (8). Inadequate resolution of the psychological distress among faculty could potentially derail the progress and reform of higher education (9).

In the field of education, the application of emotion recognition technology is not only a technological innovation but also a reflection of educational philosophy. It emphasizes the attention to students’ emotional needs during the educational process and promotes emotional communication between teachers and students. By monitoring students’ emotional changes in real time, teachers can more flexibly adjust teaching strategies to create a more supportive and responsive learning environment. Teachers’ occupational stress arises from both external objective factors and internal subjective perceptions (10). While greater external stimuli generally lead to higher stress levels, individuals with different personalities experience stress differently under similar conditions (11). External stimuli influence internal mechanisms, and individual differences in stress perception are significant (12). Understanding which personality traits affect the internal processing of occupational stress is crucial (13). Key psychological factors involved include needs and motivations, cognitive styles, personality traits, abilities, self-expectations, experiences, and psychological readiness (14).

Teachers, like all individuals, have various needs, including physiological and social needs (15). According to Maslow’s hierarchy of needs (16, 17), these needs are integral to motivation. In the context of their professional roles, teachers’ needs also involve specific aspects of material, developmental, self-esteem, and achievement needs (18–20). The process of needs and motivations is cyclical: unmet needs can lead to negative emotions such as depression and anxiety, contributing to occupational stress (21).

Emotion recognition is a significant area of research within the broader field of study (22). Despite advancements in artificial intelligence, which excel in logical reasoning, memory, and computational power (23), AI systems lack the emotional recognition capabilities inherent to human decision-making (24). Effective human–computer interaction currently demands that computers not only perform tasks but also understand and respond to users’ emotions. Innovations such as wearable devices and miniature body monitoring systems have facilitated the collection and analysis of physiological signals to gauge emotional states. These technologies are increasingly applied in various fields, including monitoring the emotional states of drivers, pilots, and medical professionals, to enhance safety and performance (25).

In the field of educational human–computer interaction, the development of emotion recognition technology is crucial for improving the quality of interaction between teachers and students. Although previous studies have made certain progress in emotion recognition, existing systems often rely on a single physiological signal, such as electrocardiography (ECG) or electromyography (EMG), which limits the accuracy and comprehensiveness of emotional state assessment. Furthermore, most existing research focuses on applications in non-educational settings, and there is still a lack of understanding of emotional dynamics in educational environments.

This study aimed to develop an objective emotion recognition system that integrates multiple physiological signals. We use wearable devices to capture electrocardiography (ECG), electromyography (EMG), electrodermal activity, and respiratory signals and assess emotional states through feature extraction, selection, and fusion techniques. The novelty of our research lies in (1) Employing a multi-physiological signal fusion method to enhance the accuracy and standardization of emotional state assessment. (2) Applying deep learning models, particularly convolutional neural networks (CNN) combined with attention mechanisms, to improve the predictive power and interpretability of the model. (3) Applying the model to real-time interactions in educational environments provides a new perspective on the application of emotion recognition technology in the field of education.

The structure of this paper is as follows: Section 1 introduces the background and significance of emotional recognition in educational human–computer interaction, highlighting the challenges faced by university teachers and the importance of addressing their mental health. Section 2 provides a theoretical basis and related concepts, including the determination of psychological crisis and the main manifestations of college teachers’ psychological states. Section 3 discusses the related technologies used in the study, focusing on feature extraction methods and the empirical mode decomposition (EMD). Section 4 presents the experimental results and analysis, including the simulation experiments and comparative analysis of different classification models. Finally, Section 5 concludes the paper by summarizing the findings and discussing their implications for the evaluation of mental health among educators and students.

## 2 Related concepts and theoretical basis

### 2.1 Related theoretical analysis

#### 2.1.1 The theory of causes of psychological crisis

The genesis of a psychological crisis is rooted in the theory that human behavior is propelled by unmet needs, which are stratified into a hierarchy. This hierarchy, as proposed by Abraham Maslow, suggests that individuals are motivated to fulfill their basic physiological needs before aspiring to higher levels of satisfaction, such as safety, love, esteem, and self-actualization. When an individual encounters barriers or setbacks in fulfilling these needs, it can lead to a range of emotional responses, including discomfort, isolation, melancholy, and a sense of hopelessness.

Human beings are also constrained by a multitude of factors, including their moral character, the socio-political landscape, the historical epoch, and the temporal conditions. In the present social context, the ongoing development of socialist values represents an incremental and perpetual historical journey. Cognitive dissonance theory, a cornerstone of sociological psychology, posits that inconsistencies between an individual’s beliefs, values, and actions can lead to psychological discomfort. If these dissonances are not addressed promptly and effectively, they can escalate into a full-blown psychological crisis.

Attribution theory, on the other hand, aids in the exploration of the reasons behind an individual’s actions and outcomes, whether they be internal factors such as personal abilities or external pressures such as environmental conditions. According to Bernard Weiner, the attributions individuals make regarding their successes and failures are influenced by their personality traits and past experiences. This theory provides a framework for understanding the causal factors that may precipitate a psychological crisis, thereby offering a starting point for intervention and resolution.

#### 2.1.2 Psychological crisis intervention theory

Psychological crisis intervention theory refers to techniques aimed at providing immediate support to individuals experiencing a crisis. The primary goal is to help individuals regain their psychological balance by mobilizing their own resources. Key components of this theory include the following: (1) Immediate support: The theory emphasizes the importance of timely assistance during a crisis, which can prevent the escalation of psychological distress. (2) Active involvement: It encourages the active involvement of individuals in their recovery process, fostering a sense of control and empowerment. (3) Structured approach: Crisis intervention typically follows a structured process that involves assessing the individual’s needs, developing coping strategies, and providing emotional support.

There are many definitions of crisis intervention. Crisis intervention is a technique to provide effective help and support to individuals or groups in crisis and to mobilize their own potential through this help and support to restore and re-establish their pre-crisis psychological balance. Zhai Shutao believes that crisis intervention is a short-term help process of caring for and supporting people who are in distress or suffering setbacks and restores their psychological balance through this process. Crisis intervention is a process in which members of society take effective measures to help individuals, families, and groups in distress so that they can survive the crisis and restore their psychological balance. Crisis intervention is to take effective measures for individuals in a state of psychological crisis to overcome the crisis and re-adapt to life. Crisis intervention is a short-term treatment that provides support and assistance to people who are experiencing a personal crisis, are in distress or are suffering setbacks, and are about to be in danger (suicide) so that they can restore psychological balance and achieve pre-crisis behavioral levels.

In the context of this study, psychological crisis intervention theory is applied to guide the development of our emotion recognition system. The theory informs our approach to identifying and responding to emotional distress in educational human–computer interactions. By integrating psychological crisis intervention principles, we aim to equip our system with the sensitivity and responsiveness necessary for immediate emotional states, thereby enhancing the effectiveness of emotional recognition in real-time educational settings.

### 2.2 Main manifestations of the psychological state of college teachers

A psychological crisis manifests across various dimensions, including cognitive, physical, emotional, behavioral, and interpersonal realms. Cognitively, an individual in the throes of such a crisis may be enveloped by sorrow, leading to alterations in memory and perception. This can result in challenges distinguishing between the nuances of experiences and a blurring of the lines between similar and dissimilar events. Decision-making and problem-solving skills are compromised, although clarity often returns swiftly once the crisis subsides. Physically, a crisis can trigger symptoms such as insomnia, headaches, exhaustion, heart palpitations, chest constriction, overall malaise, loss of appetite, and indigestion, thereby temporarily impairing bodily functions.

Emotionally, a psychological crisis can dramatically alter one’s affective state, marked by intense feelings of tension, anxiety, emptiness, and a sense of loss, often accompanied by secondary emotions such as fear, anger, guilt, distress, and shame. Anxiety is the most prevalent response, with depression being a graver outcome, and anger potentially escalating to aggressive actions. Behaviorally, the external conduct of body may shift as part of its stress response, serving as a coping mechanism to the crisis at hand. Interpersonally, during a psychological crisis, individuals may become increasingly withdrawn into themselves, becoming averse to communication, resistant to assistance or advice, unable to form trusting relationships, and consequently, experiencing isolation.

For university educators specifically, the manifestations of a psychological crisis are particularly pronounced. The emotional fluctuations experienced by teachers under stress are intricately tied to their anticipation and appraisal of stress outcomes. Successfully managing stress can lead to positive emotional experiences; however, the spectrum of emotional responses can range from anxiety and fear to depression and anger. Anxiety is the most typical reaction, with its impact on an individual’s varying environmental coping behaviors. Low levels of anxiety can slow response times and diminish productivity, while moderate levels can heighten alertness and enhance a teacher’s adaptability and stress management. In contrast, excessive or misplaced anxiety can diminish one’s capacity to adapt to environmental shifts, potentially leading to generalized anxiety that hinders effective coping mechanisms in response to environmental changes.

## 3 Related technologies

### 3.1 Feature extraction method

In emotion recognition research, which features of physiological signals are extracted has a significant impact on the classification effect. Different features will contain different information about the corresponding physiological signals.

In this study, we have established criteria for classifying emotional states based on extensive research in psychology and behavioral science as well as previous studies in the field of human–computer interaction. Specifically, our classification of emotional states is based on the following core dimensions: physiological activation level, electrodermal activity, and changes in respiratory patterns. For example, states of stress or anxiety are often accompanied by increased respiratory rate and changes in depth. Based on the characteristics of these physiological signals, we categorize emotional states into the following four categories: (1) Baseline State: In this state, individuals are relaxed and not experiencing significant emotional changes due to external stimuli. (2) Stress State: Individuals experience stress or anxiety, with physiological signals showing a higher level of activation. (3) Amusement State: Individuals exhibit higher positive emotions and physiological activation when engaged in enjoyable or pleasant activities. (4) Meditation State: During meditation or deep relaxation, physiological signals show lower activation levels and regular respiratory patterns.

**Figure 1:**
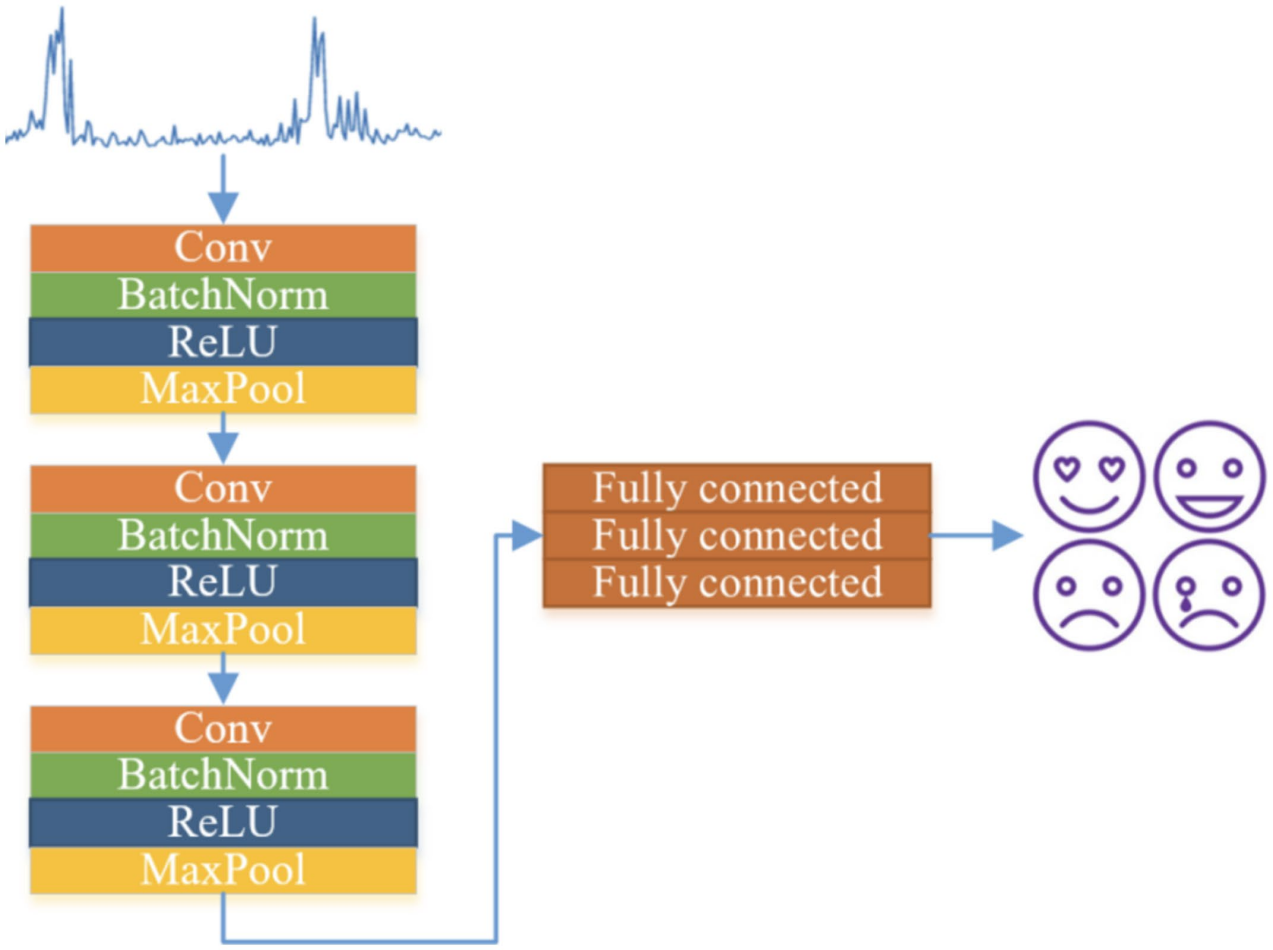
CNN-based human mental state recognition model.

In order to comprehensively study which features can contain more physiological signal features in different emotional states, as shown in Figure 1, this study uses four methods for physiological signals. Feature extraction includes time and Hilbert-Huang transform (HHT)-based features. Although different types of features can more comprehensively reflect the physiological signal changes in different emotional states, too many features are prone to feature redundancy, optimal feature set before emotion classification and recognition.

#### 3.1.1 Time-domain feature extraction

In time-domain feature extraction, the influence of emotional states on physiological signals is reflected through changes in the signal waveform. We mainly extract statistical features such as mean, mean square, first-order difference, and second-order difference, with the corresponding Equations as follows:

Mean (Equation 1):


(1) $$\mu_{x}=\frac{1}{N}\overset{N}{\underset{n=1}{\sum }}X_{n}$$

Mean Square (Equation 2):


(2) $$\sigma_{x}={\left(\frac{1}{N-1}\overset{N}{\underset{n=1}{\sum }}{\left(X_{n}-\mu_{x}\right)}^{2}\right)}^{\frac{1}{2}}$$

First-order difference (Equation 3):


(3) $$\delta_{x}=\frac{1}{N-1}\overset{N-1}{\underset{n=1}{\sum }}|X_{n+1}-X_{n}|$$

Second-order difference (Equation 4):


(4) $$\gamma_{x}=\frac{1}{N-2}\overset{N-2}{\underset{n=1}{\sum }}|X_{n+2}-X_{n}|$$

Where,

*N*: Total number of sample points. $X_{n}$ : Value of the *n*-th sample point. $X_{n+1}$ : Value of the (n + 1)-th sample point. $X_{n+2}$ : Value of the (n + 2)-th sample point.

#### 3.1.2 Time-frequency domain feature extraction

The wavelet transform is based on the short-time Fourier transform. In addition to the idea of localization, the size of the window corresponding to the window function is fixed but the shape of the window is variable. Different frequencies have different resolutions.

Discrete wavelet transform is a kind of wavelet transform that discretizes continuous wavelet transform and discretizes scale variable *a* and translation variable *b*. The Equation 5 is as follows:


(5) $$b=ka_{0}^{j}b_{0},j,k\in Z$$

Where $a_{0}$ is the scaling factor, $b_{0}$ is the translation factor, *j* and *k* are integers representing the scale and translation levels, respectively.

The discrete wavelet function is as follows (as shown in Equation 6):


(6) $$\psi_{j,k}\left(t\right)=\frac{1}{\sqrt{a_{0}^{j}}}\psi \left(a_{0}^{-j}-\mathrm{kb}\right)$$

Where *ψ* is the mother wavelet, and *b* represents scaling and translation.

The discrete wavelet transform of a function *f* (*t*) can be expressed (as shown in Equation 7):


(7) $$\lambda_{i}=\lim_{\tau \to \infty }t\left[\frac{p_{i}\left(t\right)}{p_{0}\left(t\right)}\right]$$

This equation transforms the signal into wavelet coefficients, which can then be divided into approximate and detailed coefficients.

The choice of basis functions (mother wavelets) significantly influences the decomposition results. Based on the characteristics of physiological signals, the Daubechies wavelet basis dbN is selected for decomposition in this study, with the following properties:

1. Asymmetry: most of the dbN wavelet bases are asymmetric.
2. Regularity: the regularity of the wavelet increases as *N* increases.
3. Orthogonality: the wavelet functions are orthogonal, making them ideal for signal decomposition.

Following feature extraction, our model subjects the features to a rigorous selection process to ensure optimal model performance. We employed a combination of filter, wrapper, and embedded methods to select the most informative features. Filter methods initially reduced the feature space by eliminating features with no significant statistical relationship to the emotional states. Wrapper methods, specifically a genetic algorithm, further refined the feature set by searching for the optimal combination of features that maximized predictive accuracy. Finally, embedded methods through regularized regression intrinsically performed feature selection by penalizing less important features. The criteria for feature selection included statistical relevance, redundancy elimination, and the improvement of model performance metrics such as accuracy and F1 score.

### 3.2 The theoretical basis of EMD

**Figure 2:**
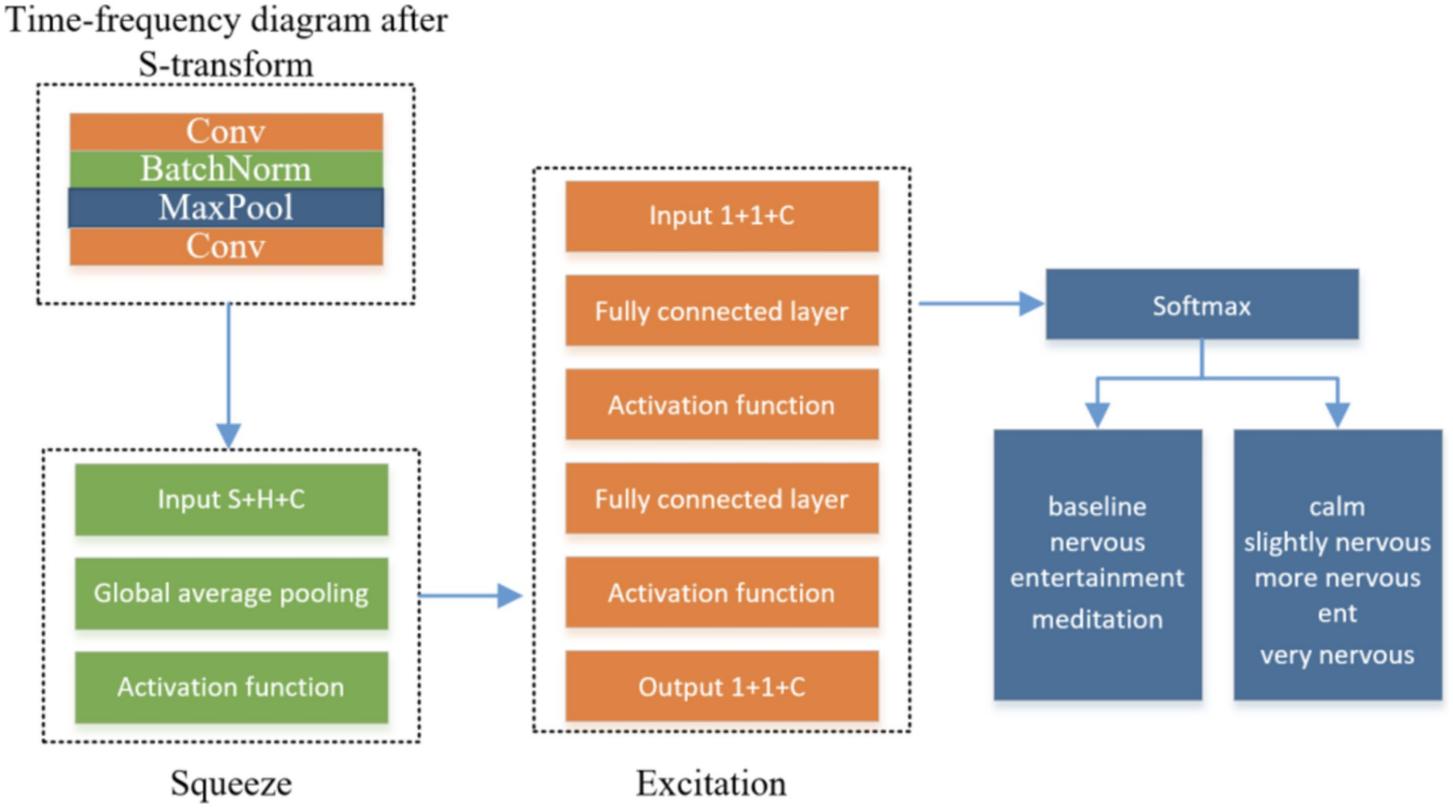
Structure diagram of SE-CNN human mental state recognition model.

Such an algorithm is to decompose the components of different frequencies in the signal, and the separated frequency components are different. These components are called features:

1. The number of zeros and poles in the IMF are equal or differ by at most 1;
2. The decomposition signal is shown in Figure 2.

Step 1: Let *u*_1_(*t*) be the signal composed of the maximum points and *u*_2_(*t*) be the signal composed of the minimum values. The mean $m\left(t\right)$ of these two signals is given by (as shown in Equation 8):


(8) $$m\left(t\right)=\frac{1}{2}\left(u_{1}\left(t\right)+u_{2}\left(t\right)\right)$$

Where $u_{1}\left(t\right)$ and $u_{2}\left(t\right)$ represent the signals composed of the maximum and minimum values of the original signal, respectively.

Step 2: Subtract the original signal sequence from Equation 9 to obtain a new sequence $h\left(t\right)$ :


(9) $$h\left(t\right)=x\left(t\right)-m\left(t\right)$$

Where $x\left(t\right)$ is the original signal sequence.

Step 3: Judge whether conditions of the IMF. If does not satisfy the conditions of IMF, it is regarded as a new $x\left(t\right)$ , and it satisfies the conditions of the IMF. In the actual calculation, too much repetition of the above process will lose the practical significance of the signal to a certain extent, so it is necessary to formulate a stopping criterion. The emotional changes of individual teachers under stress are also closely related to their prediction and evaluation of stress outcomes. Successfully coping with stressors often brings pleasant and happy emotional experiences to teachers. According to the severity of emotional changes, it can be expressed as anxiety, fear, depression, anger, and so on. Anxiety is the most common emotional response in the stress response. When the anxiety level is low, it affects the individual’s behavior in coping with the environment. The response is often slow and the efficiency of homework is not high. Moderate levels of anxiety can enhance an individual’s alertness and bolster teachers’ capacity to adapt to their environment and manage stressors effectively. Conversely, excessive or misplaced anxiety can diminish the ability to address environmental changes. Moreover, there is a risk of anxiety becoming generalized, potentially impairing teachers’ efficacy in responding to environmental shifts.

The commonly used stopping criterion is as follows (as shown in Equation 10):


(10) $$S_{D}=\overset{T}{\underset{i=0}{\sum }}{\left[\frac{h_{1\left(k-1\right)}\left(t\right)-h_{1k}\left(t\right)}{h_{1\left(k-1\right)}\left(t\right)}\right]}^{2}$$

In the equation, $S_{D}$ represents the stopping criterion, with a threshold value typically ranging from 0.2 to 0.3. When it is less than this threshold, the iteration process will be stopped.

Step 4: The IMF component is obtained as $C_{1}=h_{1k}\left(t\right)$ , and the remainder after separation is as follows (as shown in Equation 11):


(11) $$r_{1}\left(t\right)=x\left(t\right)-C_{1}$$

Where $r_{1}\left(t\right)$ is the residuals after the separation of the first IMF components from the original signal.

Step 5: Repeat the above steps to obtain subsequent IMF components $C_{2}$ , $C_{3}$ , … $C_{n}$ .

Through the above steps, the original signal composed of multiple original signals can be reconstructed by adding each IMF component and the residual. The equation is (as follows as shown in Equation 12):


(12) $$x\left(t\right)=\overset{n}{\underset{i=1}{\sum }}C_{i}\left(t\right)+r_{n}\left(t\right)$$

After the above decomposition process, we can see that the well so that we can extract the internal features of the signal, but the EMD algorithm has a problem that cannot be ignored; that is, it has modal aliasing, which will lead to the IMF obtained after decomposition loses its physical meaning. A new decomposition method for the aliasing problem is the overall empirical mode decomposition algorithm.

However, the instantaneous frequency of any time series is not always meaningful, it must meet certain conditions, which is why the signal should be EMD decomposed to obtain IMF before HHT is performed on the signal. After the signal decomposition process, the equation to reconstruct the original signal is as follows:


(13) $$s\left(t\right)=Re\overset{n}{\underset{i=1}{\sum }}a_{i}\left(t\right)e^{j\phi_{i}\left(t\right)}=Re\overset{n}{\underset{i=1}{\sum }}a_{i}\left(t\right)e^{j\int \omega_{i}dt}$$

Where $s\left(t\right)$ is the reconstructed signal after the Hilbert–Huang transform (HHT), which is the sum of the real parts of the complex exponentials representing each IMF component. $a_{i}\left(t\right)$ represents the amplitude of the ith IMF component. $\phi_{i}\left(t\right)$ represents the phase of the ith IMF component. $\omega_{i}$ represents the instantaneous frequency of the ith IMF component.

In Equation 13, Re means taking the real part. The Hilbert time spectrum $H\left(\omega ,t\right)$ is as follows:


(14) $$H\left(\omega ,t\right)=Re\overset{n}{\underset{i=1}{\sum }}a_{i}\left(t\right)e^{j\int \omega_{i}\left(t\right)dt}$$

Following Equation 14 and the process described above, we find that the Hilbert-Huang Transform (HHT) is more adaptable to the instantaneous frequency of the signal and can represent varying frequencies. This makes the HHT a more suitable tool for analyzing non-stationary signals. As shown in Equation 15:


(15) $$Z\left(t\right)=X\left(t\right)+iY\left(t\right)=a\left(t\right)e^{i\theta \left(t\right)}$$

Where $Z\left(t\right)$ is the complex form of the signal after applying the Hilbert transform, where $X\left(t\right)$ is the original signal and $Y\left(t\right)$ is the Hilbert transform of the original signal. $a\left(t\right)$ represents the amplitude of the signal $Z\left(t\right)$ . $\theta \left(t\right)$ represents the phase of the signal $Z\left(t\right)$ .

### 3.3 Analysis of model decision process

The emotion recognition model described in the study employs a multitude of physiological signals, including ECG, EMG, electrodermal activity, and respiratory signals, to create a comprehensive representation of emotional states. By fusing these diverse biological indicators, the model provides a more detailed and accurate depiction of the emotional landscape than analyses based on a single signal type.

In the feature extraction phase, the model utilizes both time-domain and time-frequency domain techniques to capture the intricacies within each physiological signal. The time-domain analysis focuses on statistical properties such as the mean heart rate and respiratory rate, while the time-frequency domain analysis uses wavelet transforms to reveal the spectral content of the signals. This dual approach offers insights into the dynamic changes associated with varying emotional states.

Following feature extraction, the model subjects the features to a feature selection process, where redundant or less informative features are eliminated. This step is essential for preventing overfitting, ensuring that the model maintains high predictive accuracy and the ability to generalize across different datasets.

The decision-making process of the model is primarily driven by a neural network structure, specifically a convolutional neural network (CNN). This structure is adept at capturing non-linear relationships between physiological features and emotional states. The layered architecture of CNN enables it to automatically learn hierarchical feature representations, where complex features are constructed from simpler ones, leading to a sophisticated understanding of the data.

To enhance the interpretability of model, attention mechanisms are integrated into the neural network. These mechanisms allow the model to focus on the most informative features during the classification process. By highlighting the most relevant aspects of the input signals, such as significant ECG peaks or variations in respiratory rates, the model not only improves its predictive capabilities but also gains the ability to explain its predictions.

This level of transparency in the decision-making process is crucial, as it allows for a deeper understanding of the predictions of the model and the physiological indicators that underpin them. It clarifies which specific changes in physiological signals are most indicative of certain emotional states, effectively demystifying the often opaque nature of neural networks.

The emotion recognition model is distinguished by its integration of multiple physiological signals, sophisticated feature extraction and selection processes, and a neural network-driven decision-making process enhanced by attention mechanisms. These elements collectively strengthen the predictive accuracy of the model and provide a level of interpretability that is vital for its practical application in real-world scenarios.

## 4 Experimental results and analysis

### 4.1 Experimental simulation

In the experiment of this paper, the public data set WESAD and the electrocardiographic signal ECG of the self-collected data set are used to build a model for the four-category recognition of the human mental state. The following is an introduction to the two data sets.

This study is based on the disclosed data set, Wearable Stress and Affect Detection Dataset (WESAD), which uses the ECG signal to identify the psychological state of the human body. The WESAD dataset is a publicly available dataset specifically designed for research on the application of wearable devices in stress and emotion detection. We chose this dataset because it provides a wealth of physiological signal data (such as electrocardiography ECG) along with corresponding psychological state labels, providing an ideal data foundation for our study on emotion recognition systems. Furthermore, the WESAD dataset includes physiological signals under various psychological states, which aids in training and validating our emotion recognition model across different emotional states. The following is an introduction to the acquisition signals and experimental procedures of the WESAD dataset. The public data set contains the physiological signal data and mental state labels of 15 subjects, 12 men and 3 women. The average age of the subjects is 27.5 years old, and their personalized physiological characteristics such as height, age, gender, and weight record it. Psychological crisis can be manifested in cognitive, physical, emotional, behavioral, interpersonal, and other aspects. In terms of cognition, when an individual is in a psychological crisis, his body and mind are immersed in grief, which will lead to changes in his memory and perception, manifested as difficulty in distinguishing the similarities and differences between things, and ambiguous relationships between things experienced.

Initially, we performed denoising on the raw physiological signal data to eliminate outliers caused by sensor malfunctions or improper user operation. Subsequently, we applied band-pass filtering to the signals to remove high-frequency noise and low-frequency drift, ensuring the quality of the signals. To facilitate analysis, we segmented the continuous physiological signal data into fixed-length time windows. For the WESAD dataset, we chose a 10-s time window, allowing us to capture short-term changes in emotional states. Some signals underwent baseline correction to eliminate long-term trends and baseline drift in the physiological signals. During the preprocessing, we removed data with poor signal quality or unclear labels. We believe that such data could affect the training effectiveness of the model and the accuracy of classification. Additionally, we excluded cases with too few samples in specific psychological states to ensure that each category has sufficient data to support model learning.

**Figure 3:**
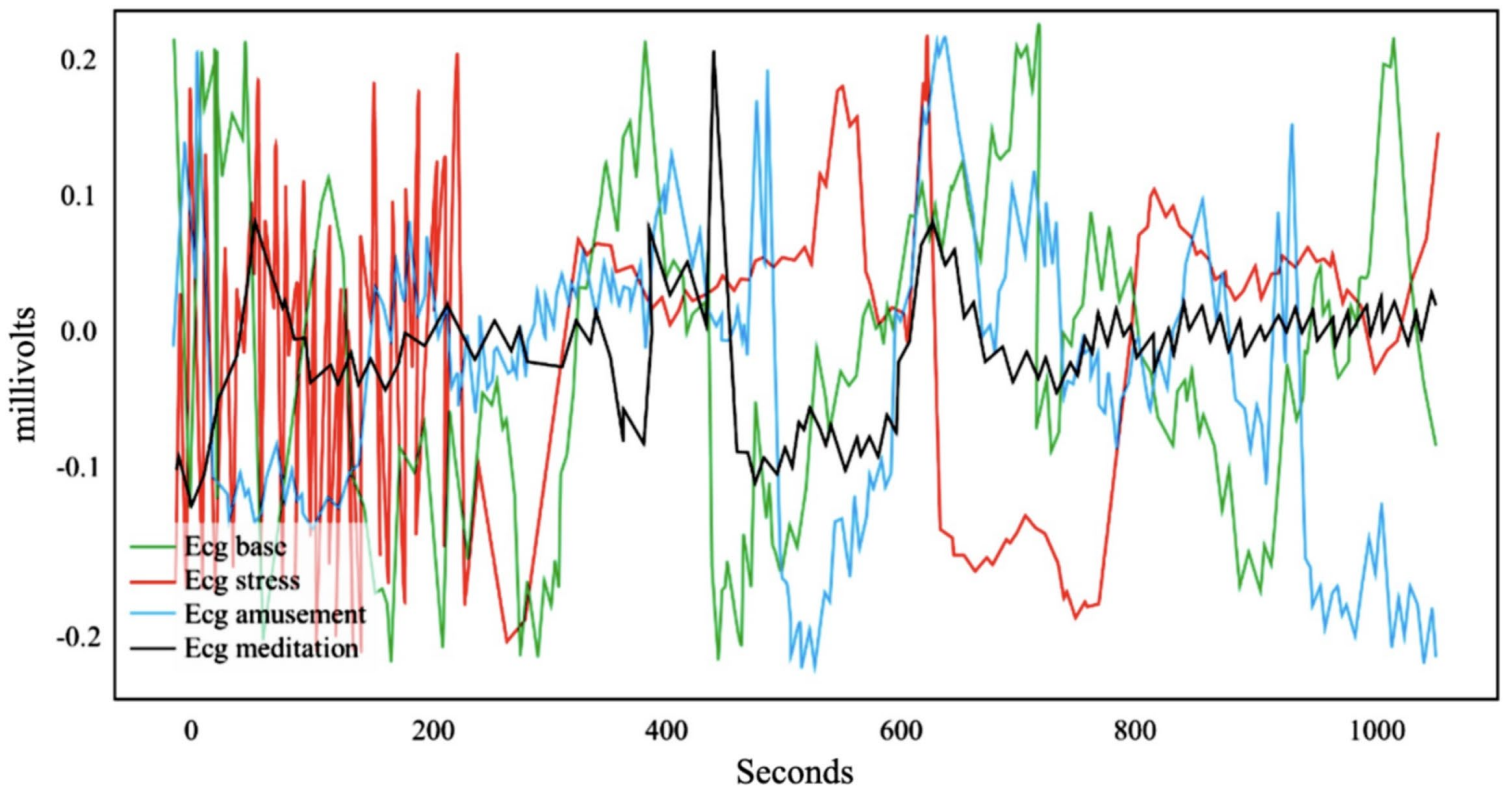
Data waveform of ECG signal in WESAD.

Figure 3 shows the ECG signal data waveform of an experimental subject in WESAD under different experimental conditions. It can be seen intuitively that the ECG signal waveform characteristics of the four different psychological states of baseline, amusement, stress, and meditation are quite different. Among which is the waveform of the ECG meditation state. Compared with the baseline, the ECG signal in the entertainment state has a steeper slope. This also proves the rationality of human mental state recognition through ECG signals to a certain extent.

The readme.txt of the WESAD data set records the age, gender, height, weight, and other personal information of the subjects; the quest questionnaire file provides the subjects’ subjective scores on their psychological state under the experimental process at each stage at that time, using PANAS, SSSQ and other personal assessment questionnaires; the pkl file provides all physiological signal data and corresponding labels of the two devices of the experimental subjects. This paper chooses to collect and preprocess ECG signal data based on the pkl file corresponding to each experimental object because the tags and data in the pkl file are stored in an array, which is convenient to establish one-to-one corresponding data and tag pairs.

**Figure 4:**
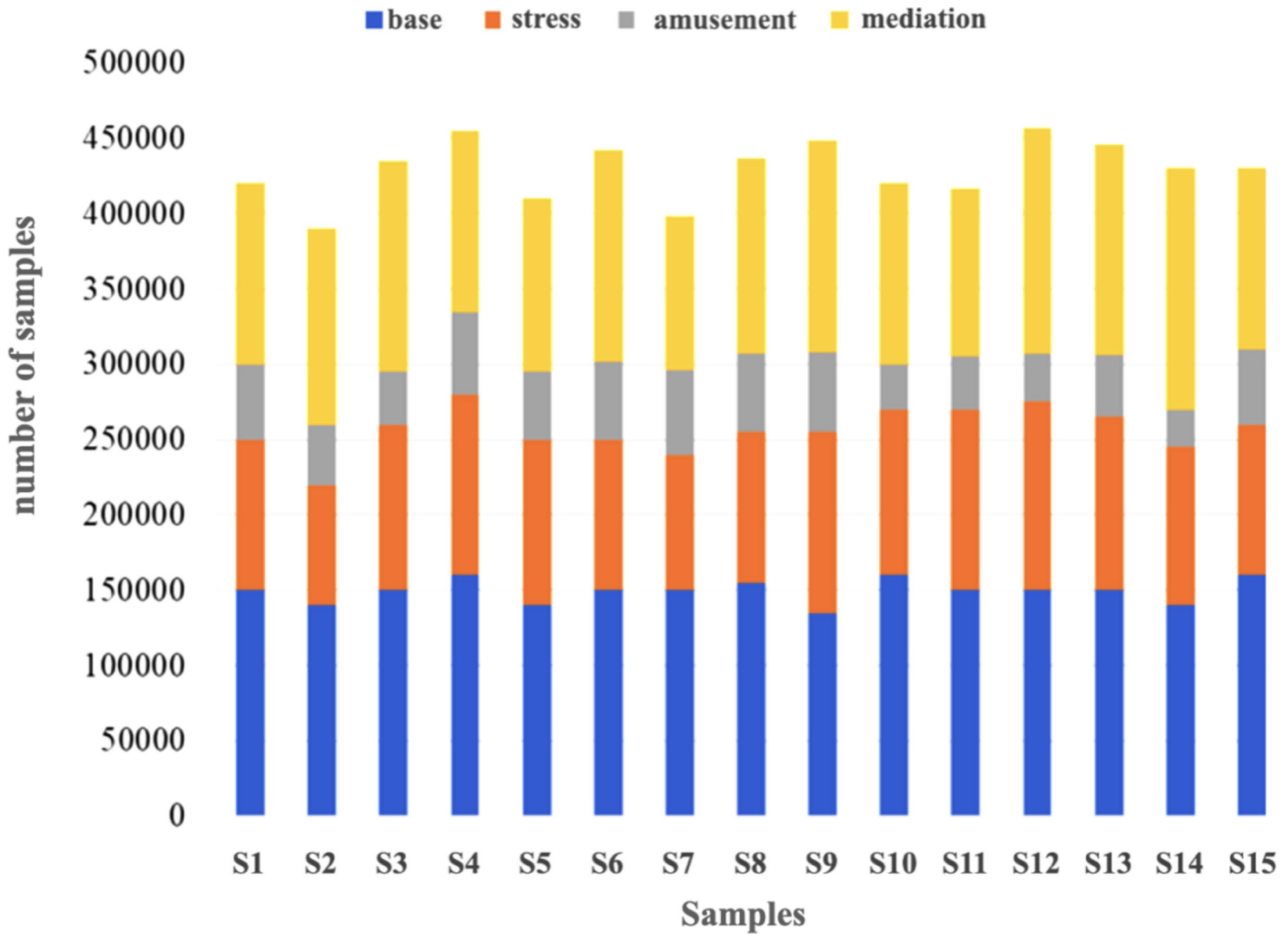
The amount of data for each mental state of ECG signals in WESAD.

Through the settings of the above different experimental procedures and conditions, four different psychological states of the experimental subjects were stimulated, which provided the data basis for the construction of the human psychological state recognition model in this paper. The corresponding psychological state labels are 1, 2, 3, and 4, respectively. The data volume and data distribution are shown in Figure 4.

Many previous experiments utilizing the WESAD dataset have focused on recognizing two-category (baseline and nervous) or three-category (baseline, nervous, and entertainment) mental states, often omitting the meditation state. This oversight is primarily due to the similarities in ECG signal waveforms between the meditation, entertainment, and baseline states, which can lead to significant confusion during identification. Consequently, expanding the classification to include four categories, based on the original three, is likely to result in some decrease in accuracy due to the inherent similarities in signal characteristics. It is important to note that signals carry distinct meanings. While the academic community has not yet reached a consensus on the definition of a psychological crisis, the perspectives mentioned above convey essentially the same concept. We interpret a psychological crisis as a state of psychological disequilibrium that arises in response to crisis events.

Beyond merely denoising the data, physiological signals from the experimental subjects must be segmented into time windows before mental state feature extraction can occur. The chest device ECG signals of WESAD dataset, used in this paper, have a sampling frequency of 700 Hz. For ease of processing, these signals are divided into fixed-size time windows, with this study considering 10-s intervals of continuous data as a single sample, equating to 7,000 data points. The self-collected dataset, MSSFT, has a sampling frequency of 125 Hz and is processed using the same time window approach, yielding 1,250 data points per sample.

In addition to the WESAD dataset, our model was also tested on the self-collected MSSFT dataset. The MSSFT dataset contains ECG signals from participants with diverse backgrounds, providing us with an opportunity to assess the model’s generalization capabilities. Similar to the WESAD dataset, we applied the same preprocessing steps to the ECG signals in the MSSFT dataset, including denoising, band-pass filtering, and segmentation into fixed-length time windows. The MSSFT dataset has a sampling frequency of 125 Hz, and we also used a 10-s time window, with each sample containing 1,250 data points. The experimental results, as shown in Table 1, indicate that our model also demonstrated the ability to recognize different psychological states on the MSSFT dataset, although the accuracy was slightly lower than on the WESAD dataset. This may be attributed to differences in data distribution and noise levels between the two datasets. These results not only suggest that our model has some applicability on new datasets but also highlight the need to further improve the generalization capabilities of model.

**Table 1:** Performance comparison of the model on WESAD and MSSFT datasets.

Metric	WESAD dataset	MSSFT dataset
Accuracy	87.90%	85.50%
Precision	88.30%	86.20%
Recall	87.71%	85.80%
F1 Score	87.71%	85.65%

### 4.2 Comparative analysis of three-category human mental state models

To comprehensively evaluate the performance of our model, we employed a variety of evaluation metrics, including accuracy, precision, recall, and F1 score. These metrics are calculated by comparing the predicted outcomes of the model with the actual labels. Accuracy represents the proportion of samples that the model predicted correctly out of the total samples; precision represents the proportion of samples predicted as positive that are actually positive; recall represents the proportion of actual positive samples that are correctly predicted as positive by the model; the F1 score is the harmonic mean of precision and recall, used to measure the overall performance of the model. Furthermore, to assess the stability and generalizability of our model, we used 5-fold cross-validation. This method involves dividing the dataset into five equal parts, using four parts for training and the remaining part for testing in each iteration, repeating this process five times, and calculating the average performance metrics.

**Figure 5:**
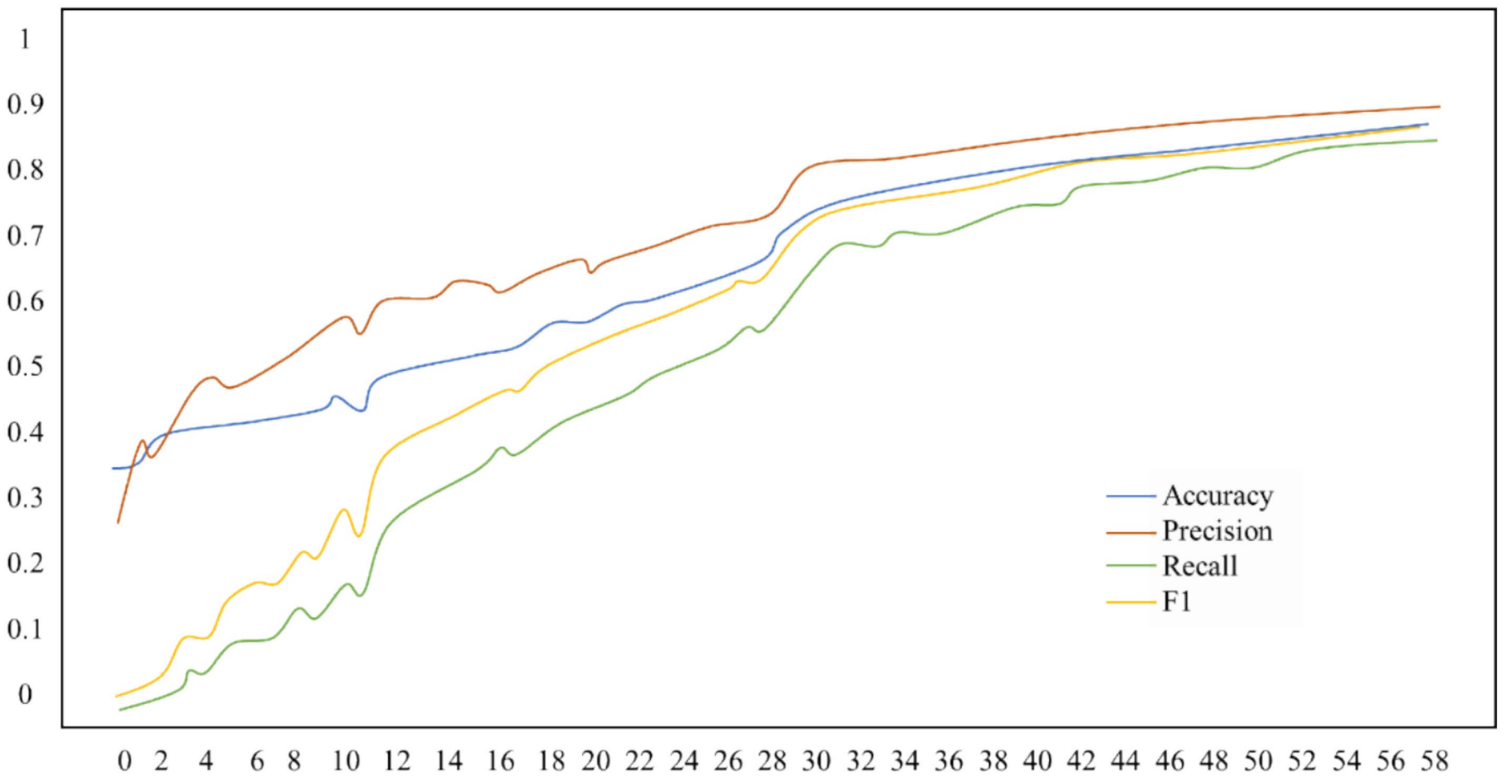
Four classification results of the multi-level two-channel fusion model (parallel) in the WESAD dataset.

Four classifications (baseline, nervousness, entertainment, and meditation) are implemented on the WESAD public data set, and the epoch is set to 60. The loss line graph and the accuracy, precision, recall, and F1 score line graph are shown in Figure 5. The accuracy is 87.90%, with an F1 score of 87.71%.

**Figure 6:**
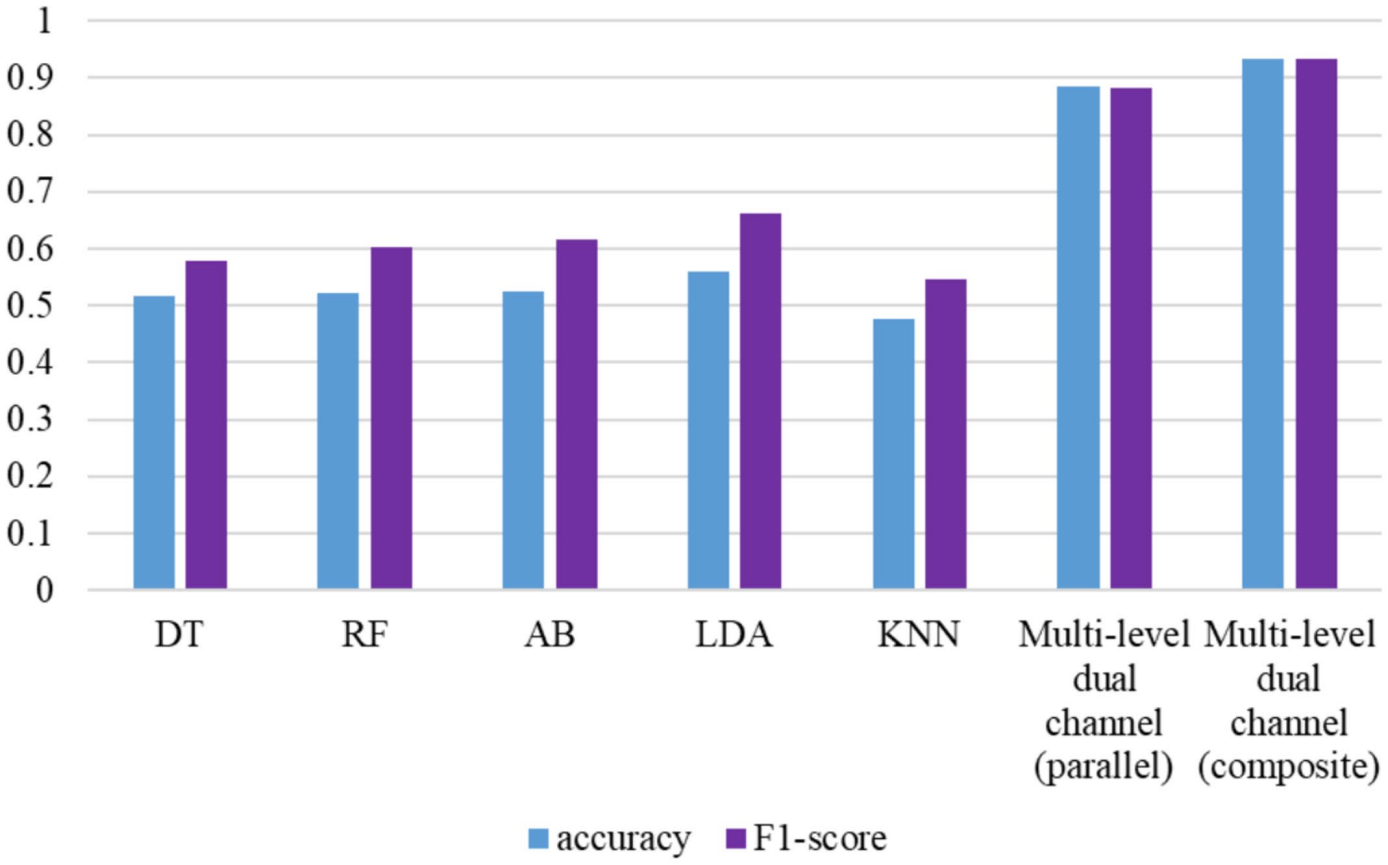
Comparison of the results of three-category recognition of mental states based on WESAD.

Given that the source publication of WESAD dataset already presents a three-category mental state recognition model—differentiating among baseline, entertainment, and nervous states using ECG signals—this paper refrains from replicating that experiment. Instead, we directly reference the outcomes of the aforementioned model to benchmark against the novel methods introduced in this study. The methodologies outlined in the original text encompass a multi-level dual-channel fusion human mental state recognition model (parallel) and a multi-level dual-channel fusion model (composite), both of which are developed in this chapter. A comparative analysis of results of these methods is depicted in Figure 6, showcasing the initial five model outcomes constructed in this chapter.

According to the above figure, it can be concluded that compared with the original results, the multi-level dual-channel fusion human mental state recognition model proposed in this chapter can better identify the three mental states of baseline, entertainment, and nervousness on the WESAD data set. The effectiveness of the method can be used to further refine the classification of mental states. The following experiments will pay more attention to the study of the four classifications.

### 4.3 Comparison and analysis of experimental model results

To compare the performance differences between different model configurations, we conducted a statistical significance analysis using paired *t*-tests. The significance level was set at 0.05. By calculating the correlation coefficients between features and performing *t*-tests, we assessed the changes in physiological signal features across different emotional states. These analyses indicate that the differences in emotional states identified by our model are statistically significant, further validating the effectiveness and reliability of our model.

**Figure 7:**
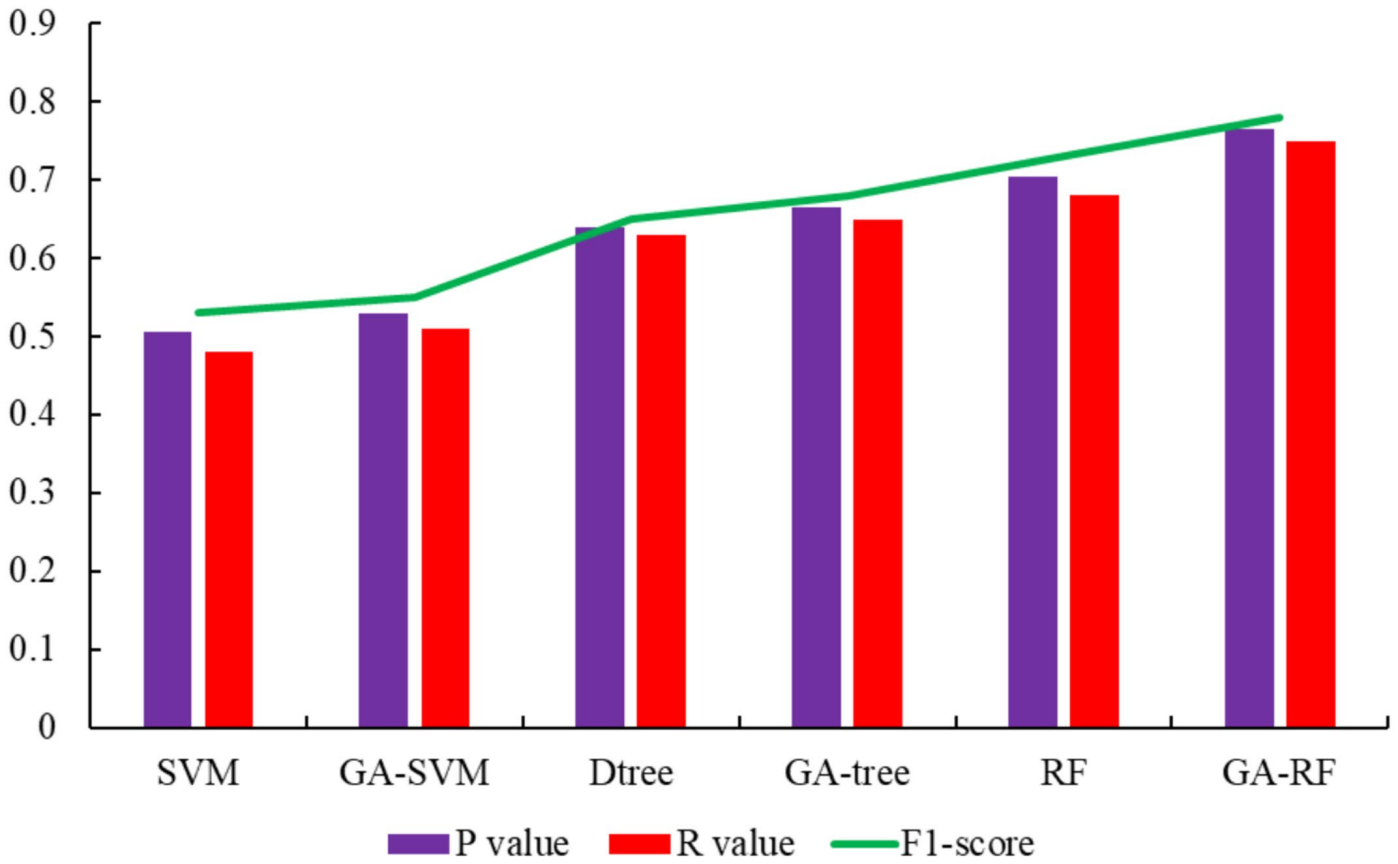
Evaluation of different classification models for internal and external tendencies.

In the 1,303 samples, the results of the internal and external propensity classification model in the GA-RF are shown in Figure 7.

**Figure 8:**
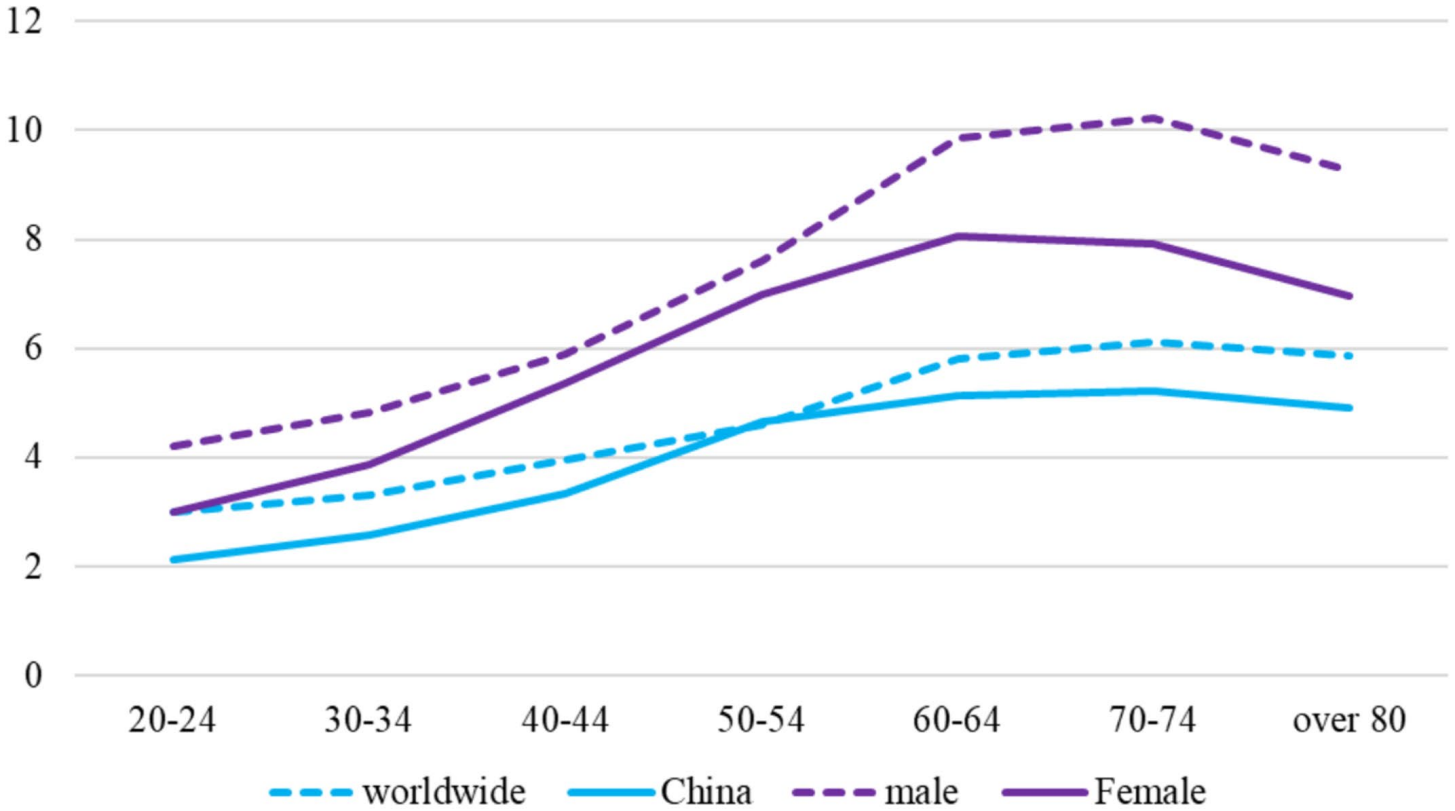
Evaluation of different models for two-category depression.

Figure 8 illustrates the comparative analysis between the original model group, which includes Decision-Tree, SVM, and RF, and the control group that incorporates the genetic algorithm for feature extraction, including GA-Decision-Tree, GA-SVM, and GA-RF. It is observed that the models augmented with the genetic algorithm demonstrate improved performance, suggesting that the feature extraction process of genetic algorithm enhances the representativeness of the data across each feature dimension. Specifically, the ability of genetic algorithm to refine the internal and external tendency labels results in eight parameters that are more indicative of the depressive psychological state. This enhancement is particularly evident when examining the outcomes from the 1,433 samples, where the accuracy of the two-class depression model is significantly elevated in the control group models that leverage the genetic algorithm.

Figure 8 presents the results, demonstrating that the F1 scores of the various model algorithms exceed 0.6, with the optimal GA-RF model achieving an F1 score of 0.81. This indicates that it is indeed possible to predict students’ depressive psychological states with a significant correlation to their online behavioral characteristics. By leveraging these characteristics, we can achieve real-time monitoring of depressive psychological states of students. Furthermore, when comparing the performance of models trained with features extracted by a genetic algorithm versus those trained solely on original data features, it becomes evident that the genetic algorithm-enhanced models exhibit a higher degree of reliance on specific online behaviors, such as WeChat dependency, the regularity of map website usage, and the degree of game dependence.

## 5 Conclusion

This study developed an emotion recognition system that integrates multiple physiological signals—ECG, EMG, electrodermal activity, and respiratory signals—to enhance emotional state assessment in educational human–computer interaction. The proposed model achieved an accuracy of 87.90% and an F1 score of 87.71% in classifying four emotional states: baseline, nervousness, entertainment, and meditation. These results indicate a significant improvement over existing models, particularly in handling the complexities of emotional state recognition. The combination of feature extraction and selection with a convolutional neural network architecture enabled the model to effectively capture the non-linear relationships between physiological signals and emotional states. Moreover, the integration of attention mechanisms significantly improved the interpretability of model, highlighting which features—such as ECG peaks and variations in respiratory rate—played the most crucial role in emotion prediction.

This study provides educators and educational technologists with a powerful tool for understanding and responding to the emotional states of students and teachers in real time. By providing a more accurate and objective assessment of emotions, the system can transform the educational experience, making it more personal and supportive of individual emotional needs. However, the study has limitations, including a relatively small dataset, which may restrict the model’s generalizability. Future research should focus on expanding the dataset to include a more diverse population and exploring additional physiological signals to improve accuracy. Implementing the model in real-time educational settings will also be crucial for assessing its practical effectiveness. In addition, we will employ more advanced data visualization techniques in future studies to enhance the interpretability of our findings.

The development of emotion recognition technology may have profound implications for educational policies and practices. Policymakers need to consider how to integrate this technology into the education system while ensuring that it does not exacerbate educational inequality. In practice, teachers can use emotion recognition technology to assess students’ learning motivation and engagement, thereby designing more effective teaching activities.

## Data Availability

The original contributions presented in the study are included in the article/supplementary material, further inquiries can be directed to the corresponding author.
